# Supplementary material for: Dental Disease in Rabbits (Oryctolagus cuniculus) and Its Risk Factors—A Private Practice Study in the Metropolitan Region of Chile
Source: Animals (Basel). 2023 Feb 15;13(4):676. doi: 10.3390/ani13040676 (PMC9951937; doi:10.3390/ani13040676)
Supplement: Supplementary file 1 [file animals-13-00676-s001.zip › animals-2195400-supplementary.pdf]

Supplementary material

Table S1. Classification of rabbit breeds used in the present study (Own elaboration, based on previous data [17-19]).

Breed	Adult weight	Other features
D[1]	<2kg	• Round and compact bodies<br>• Short noses, small ears, and a disproportionately large head size compared to body size, narrow in shape.<br>• Small and short limb bones.
Md[2]	2-5 kg	• Longer bodies.<br>• Longer noses, larger ears, head size proportional to body size, oval in shape.<br>• Wider and longer limb bones.
L[3]	>5kg	• Giant and robust bodies.<br>• Wide and elongated head, with long ears (>15cm).<br>• Larger and stronger limb bones.<br>• Dense fur.

D[1]= Dwarf, Md[2]= Medium, L[3]= Large.

Table S2. Frequency measurements for qualitative variables in patients diagnosed with ADD in a private veterinary clinic between the years 2018-2021.

Variable	Class	Categories	AF[1]	RF[2]
Location	1	C[3]	200	0.554
	2	I[4]	93	0.258
	3	P[5]	68	0.188
Sex	1	Female	138	0.382
	2	Male	223	0.618
Reproductive state	1	Castrated	64	0.177
	2	Intact	297	0.823
Race	1	Dwarf	105	0.291
	2	Large	12	0.033
	3	Medium	244	0.676
Body Condition Score	1	1	1	0.003
	2	2	47	0.130

	3	3	259	0.717
	4	4	48	0.133
	5	5	6	0.017
Type of housing	1	Caged	38	0.105
	2	Mixed	129	0.357
	3	Free range pets	194	0.537
Housing regimen	1	Indoor	267	0.740
	2	Mixto	53	0.147
	3	Outdoor	41	0.114
Hay	1	Absent	72	0.199
	2	Present	289	0.801
Pellet	1	Absent	15	0.042
	2	Present	346	0.958
Vegetables	1	Absent	80	0.222
	2	Present	281	0.778
Fruits	1	Absent	106	0.294
	2	Present	255	0.706

AF[1]= Absolute frequency, RF[2]= Relative frequency, C[3]= Cheek teeth, I[4]= Incisors, P[5]= All dental pieces.

Table S3. Clinical signs of patients with ADD in a private veterinary clinic between the years 2018-2021

Clinical signs	Affected system	AF
Gut stasis	G[1]	1
Anorexia	G	27
Hypomotility	G	27
Hyporexia	G	57
Diarrhea	G	1
Sialorrhea	G	7
Halitosis	G	2
Abscesses	D[2]	33
Perianal dermatitis	D	17
Hirsute fur	D	34
Cheyletiellosis	D	31
Myiasis	D	2
Fleas	D	2

Otitis	D	17
Mange	D	2
Head tilting	N[3]	3
Rolling	N	1
Epiphora	O[4]	81
Sneezing	O	25
Nasal discharge	O	17
Conjunctivitis	O	3
Ocular dermatitis	O	1
Exophthalmos	O	6
Dacryocystitis	O	29
Ocular proptosis	O	3
Bruxism	N	3
Depression	N	12
Nistagmus	N	2
Lethargy	N	1

G[1]= Gastrointestinal, D[2]= Dermatologic, N[3]= Neurological, O[4]=Ocular.
